# Machine learning prediction of conduct problems in children using the longitudinal ABCD study

**DOI:** 10.1111/jcpp.70057

**Published:** 2025-10-12

**Authors:** Kathryn Berluti, Paige Amormino, Alexandra Potter, Safwan Wshah, Abigail Marsh

**Affiliations:** ^1^ Department of Psychology Georgetown University Washington DC USA; ^2^ Department of Psychological Science University of Vermont Burlington VT USA; ^3^ Department of Computer Science University of Vermont Burlington VT USA

**Keywords:** Conduct disorder, conduct problems, machine learning, ABCD study

## Abstract

**Background:**

Children with conduct problems are at elevated risk for negative psychosocial, educational, and behavioral outcomes. Identifying at‐risk children can aid in providing timely intervention and prevention, ultimately improving their long‐term outcomes. There is a need to develop screening tools to better identify at‐risk children who may benefit from early intervention.

**Methods:**

Data were collected from the longitudinal Adolescent Brain Cognitive Development (ABCD) Study. Children completed a baseline visit at age 9–10, then returned annually for 3 years (*n* = 3,517). We used machine learning classifiers (logistic regression, Naïve Bayes, support vector machine, and random forest) to predict conduct problems (i.e., conduct disorder or oppositional defiant disorder) in children after 1, 2, and 3 years.

**Results:**

The best‐performing model (the random forest classifier) predicted children at risk for conduct problems with an accuracy of 90% or greater (AUC = 0.98 at 1 year, AUC = 0.97 at 2 years, AUC = 0.97 at 3 years). A random forest classifier simplified to include only 10 features was able to predict conduct problems nearly as well (AUC = 0.97 at 1 year, AUC = 0.96 at 2 years, AUC = 0.97 at 3 years).

**Conclusions:**

Using factors previously linked to conduct problems, we built machine learning models to identify predictors of conduct problems in children over a 3‐year period. A small number of self‐report features can be used to predict persistent conduct problems with 90% or greater specificity and sensitivity up to 3 years after initial assessment. This suggests that parent and child self‐report data, along with machine learning, can identify children at risk for persistent conduct problems.

## Introduction

Conduct disorder and oppositional defiant disorder are disruptive behavior disorders characterized by rule‐breaking, hostility, aggression, and other social and moral violations (American Psychiatric Association, 2022). Children diagnosed with these disorders are described as having conduct problems, which occur in roughly 5.7% of children, and are associated with a higher lifetime risk for psychopathology, poor academic outcomes, substance abuse, and criminal behavior (Bevilacqua, Hale, Barker, & Viner, 2018; Colman et al., 2009; Polanczyk, Salum, Sugaya, Caye, & Rohde, 2015). Predicting if a child will develop persistent conduct problems is challenging due to the large and disparate set of factors that can increase risk (Fairchild et al., 2019; Noordermeer, Luman, & Oosterlaan, 2016). However, approaches using machine learning (Chan et al., 2022) have identified developmental features predictive of conduct disorder with 91.18% accuracy using 52 features that include neighborhood, family, neurocognitive, and resting‐state functional magnetic resonance imaging (rs‐fMRI) risk factors. We aim to extend past efforts by including a more comprehensive list of features, identifying risk for conduct problems (rather than just conduct disorder), creating a simplified classifier, comparing the efficacy of four different machine learning models, and predicting conduct problems across 3 years.

Many risk factors for conduct problems can be identified before a child is born. Parental or family history of psychopathology, particularly externalizing disorders, is associated with persistent conduct problems and increased risk of criminal behavior (Murray & Farrington, 2010). This risk reflects both inherited and environmental factors, similar to other childhood psychological disorders (Hettema, Prescott, Myers, Neale, & Kendler, 2005; Ou, Liu, Shen, Xia, & Zhao, 2019; Viding & McCrory, 2012).

Early risk factors can influence the development of brain regions associated with conduct disorder, which can then lead to differences in cognitive outcomes (Fairchild et al., 2019; Noordermeer et al., 2016; Rogers & De Brito, 2016). For example, atypical development of structures, such as the caudate, putamen, and amygdala in children with conduct problems may lead to struggles responding to reward and punishment cues, which can lead to poor decision‐making, especially when compounded with reduced executive functioning (Blair, Leibenluft, & Pine, 2014; Blair, Veroude, & Buitelaar, 2018). Atypical development of structures, such as the amygdala, thalamus, and fusiform, which are involved in responding to distress cues (Fairchild et al., 2019; Noordermeer et al., 2016), may additionally impair the development of socio‐affective processes like empathy in response to others' distress (Fairchild et al., 2019; Frick, 2014). As childhood progresses, children at risk for conduct problems also exhibit behaviors that affect their peer relationships and peer acceptance, further heightening the risk for conduct problems (Fairchild et al., 2019; Moffitt, 1993; Murray & Farrington, 2010). Other risk factors in childhood include exposure to community violence, school conflict, and low school engagement (Fairchild et al., 2019; Murray & Farrington, 2010).

Considering these factors together, we employed a data‐driven approach in which we evaluated four machine learning techniques (logistic regression, Naïve Bayes, support vector machine, and random forest) to predict conduct problems in children 1, 2, and 3 years post‐initial assessment. We then pinpointed the 10 most critical features to determine whether a simplified model could maintain accurate predictions of conduct problems. We also evaluated whether a model incorporating solely brain structure and function features remained robust. As our aim was to utilize a data‐driven method to pinpoint a concise classifier capable of reliably predicting conduct problems in children over 3 years, we did not establish specific hypotheses regarding feature importance or the most robust classifier.

## Methods

### Participants

Participants included children who took part in the Adolescent Brain Cognitive Development (ABCD) Study and completed baseline, year 1, year 2, and year 3 visits. The ABCD is a large, longitudinal, multi‐site study that has recruited 11,875 children ages 9–10 and will track their development until they are 19–20 years old. Data are being collected from 21 geographically distributed research study sites across the United States. Participants were recruited through public and private elementary schools based on gender, race and ethnicity, socioeconomic status, and urbanicity to achieve a semi‐representative national sample (Garavan et al., 2018). During research visits, children and guardians complete a battery of self‐report questionnaires aimed at measuring mental health, social relationships, emotional development, cognitive capacities, and environmental factors (among many other variables). Children also complete biological measures including brain imaging (structural and task‐based functional MRI). Institutional review boards at each research site approved all study procedures. A parent or legal guardian provided written informed consent, and children provided written assent.

Because the ABCD study includes siblings, we randomly selected only one child from each family to ensure independence of data points. Only participants with complete conduct problems data at each time point and with MRI data passing the ABCD study quality check were included. More information on quality control of brain imaging measures is provided in an overview of the ABCD study MRI processing protocol (Hagler et al., 2019). This resulted in a final sample of 3,517 children (Table 1).

**Table 1:** Demographic information

Variable	Mean (*SD*)	*n* (%)
Age at baseline (months)	120.05 (7.4)	
Biological sex at birth		
Female		1,694 (48.17%)
Male		1,823 (51.83%)
Race/Ethnicity		
Asian		86 (2.45%)
Black		278 (7.9%)
Hispanic		619 (17.6%)
White		2,183 (62.07%)
Other		351 (9.98%)
Baseline diagnosis		
No diagnosis		3,272 (93.03%)
CD diagnosis		65 (1.85%)
ODD diagnosis		91 (2.59%)
Comorbid diagnoses		89 (2.53%)
1‐Year diagnosis		
No diagnosis		3,283 (93.35%)
Diagnosis		234 (6.65%)
2‐Year diagnosis		
No diagnosis		3,285 (94.07%)
Diagnosis		207 (5.93%)
3‐Year diagnosis		
No diagnosis		3,314 (94.23%)
Diagnosis		203 (5.77%)

### Measures

From the available baseline measures, a total of 51 features were selected for inclusion in the classifiers (not all measures were collected at all timepoints; only measures included at baseline were considered for inclusion), including covariates (e.g., data collection site). Biological, environmental, and psychological measures selected for inclusion were drawn from the results of prior longitudinal analysis (Moffitt, 2018) and research reviews (Fairchild et al., 2019; Frick, 2014; Moffitt, 1993; Murray & Farrington, 2010; Noordermeer et al., 2016; Rogers & De Brito, 2016) and reflected those variables most consistently associated with early‐onset, persistent behavior problems in children.

Our primary outcome variables were conduct disorder and oppositional defiant disorder symptoms assessed using the parent‐completed Child Behavior Checklist (CBCL) (Achenbach & Rescorla, 2001). This measure captures a range of developmental psychopathology symptoms, including behavioral, emotional, and social problems. We selected the conduct disorder and oppositional defiant disorder subscales. Children with standardized T‐scores at or above the “borderline clinical” (≥65) range on the conduct disorder or oppositional defiant disorder scales were labeled as having conduct problems. Scores were calculated for baseline, year 1, year 2, and year 3. Baseline scores were included as a covariate (see Supporting Information). Year 1, 2, and 3 scores were the primary outcome variables of interest.

#### Neural structure and function features

Neural structure and function features included 21 variables related to brain activation and volume at baseline collected using structural MRI and fMRI. We chose regions of interest (ROI) shown in prior reviews and meta‐analyses to exhibit differences in volume in children with versus without conduct problems (Fairchild et al., 2019; Noordermeer et al., 2016; Rogers & De Brito, 2016). ROIs selected for inclusion were: right and left amygdala, rostral anterior cingulate cortex, caudal anterior cingulate cortex, fusiform gyrus, thalamus, and medial orbitofrontal cortex. Total intercranial volume was also included as a covariate (see covariates below).

Brain volume data were collected using structural MRI, and the volume of cortical and subcortical regions was extracted using a standardized processing pipeline (Hagler et al., 2019). Reconstruction and segmentation were performed using FreeSurfer with tools validated for use in children (Ghosh et al., 2010). Subcortical ROIs were identified using an atlas‐based volumetric segmentation procedure (Fischl et al., 2002), and cortical ROIs were identified by registering them to the atlas using cortical folding patterns (Fischl, Sereno, Tootell, & Dale, 1999) and Bayesian classification rules (Desikan et al., 2006; Destrieux, Fischl, Dale, & Halgren, 2010; Fischl et al., 2004). More information about structural MRI analysis and brain segmentation is reported in an overview of the MRI analysis pipeline (Hagler et al., 2019).

Functional ROIs were selected based upon prior evidence that youth with conduct problems show atypical responses in these regions when viewing emotional stimuli (Alegria, Radua, & Rubia, 2016; Berluti, Ploe, & Marsh, 2023; Dugré et al., 2020; Noordermeer et al., 2016). Selected regions were: left and right amygdala, thalamus, fusiform gyrus, caudate, and putamen. Functional activation in these ROIs was selected from the emotional “n‐back” task. Mean beta‐weights during the n‐back task were generated and the activation difference from the fearful versus neutral face contrast was calculated (see Supporting Information). More information about the task and scan protocol is provided in an overview of the ABCD study imaging procedures (Casey et al., 2018).

#### Familial and environmental features

Familial and environmental features included variables related to prenatal events, family mental health history, community environment, and social relationships. Specifically, parent history of psychiatric conditions, prenatal alcohol and drug exposure, total number of friends and close friends, access and exposure to drugs and alcohol, neighborhood safety, school environment, school involvement, school disengagement, and adverse childhood experience (ACEs) scores were all included as familial and environmental features. More details about familial and environmental feature measurement and scoring can be found in the Supporting Information.

#### Psychological features

Psychological features included variables related to cognitive and psychosocial traits linked to conduct problems. These included picture vocabulary, oral reading recognition, list sorting, working memory, impulsiveness, and prosocial behavior. More details about psychological feature measurement and scoring can be found in the Supporting Information.

#### Demographic features and covariates

Biological sex at birth, race/ethnicity, total combined family income, and age were included as demographic features. Scanner ID, data collection site, total intercranial volume, baseline Conduct Disorder diagnosis, and baseline oppositional defiant disorder diagnosis were included as covariates in all classifiers (see Supporting Information for scoring information).

### Analysis

We aimed to identify which of four algorithms would be the most predictive of conduct problem risk over time. We compared four approaches: logistic regression, Naïve Bayes, support vector machine, and random forest classifiers, to classify children as having conduct problems after 1, 2, and 3 years following baseline testing.

For the Naive Bayes classifier, we trained a Gaussian Naive Bayes algorithm that utilizes a Gaussian distribution for feature likelihood. For the support vector machine, we used regularized linear models, stochastic gradient descent learning, and Elastic Net regularization. We also used a random grid search technique for hyperparameter tuning from 50 random searches (Bergstra et al., 2012). Parameters were set at an alpha of 0.0114, with an l1 regularization ratio of 0.1903. Finally, for the random forest, we used bootstrapped samples to build each tree to minimize the risk of overfitting.

Then, after comparing the effectiveness of the classifiers, we considered whether using the most effective classifier with a reduced number of features (i.e., only the most important features based on feature importance scores) would be comparably predictive to the full classifier. (Less important features, meaning features with smaller importance scores, are often removed from classifiers.) This approach can be clinically important because it reduces the number of features needed to predict clinically relevant outcomes. We thus created a simplified classifier using only the top 10 most important features. Finally, we investigated whether a brain‐based classifier would be comparably predictive when compared with the larger classifier to assess if brain imaging measures can provide critical information for diagnoses.

#### Missing data imputation

From the full sample of 3,517, a total of 2,700 participants were missing data. However, most of these participants (2,108) were missing only a single measure, with only 2.76% of the total data missing. Therefore, to retain all participants and reduce bias that could be introduced by excluding participants with missing data, we imputed missing data (White et al., 2011) using multiple imputations by chained equations (MICE). Due to the imbalanced nature of the dataset, we resampled the data to create more equal classes using the sampling algorithm SMOTE‐ENN (see Supporting Information for more information) (Batista, Prati, & Monard, 2004). This allowed us to retain the original 3,517 participants.

#### Feature selection

**Figure 1:**
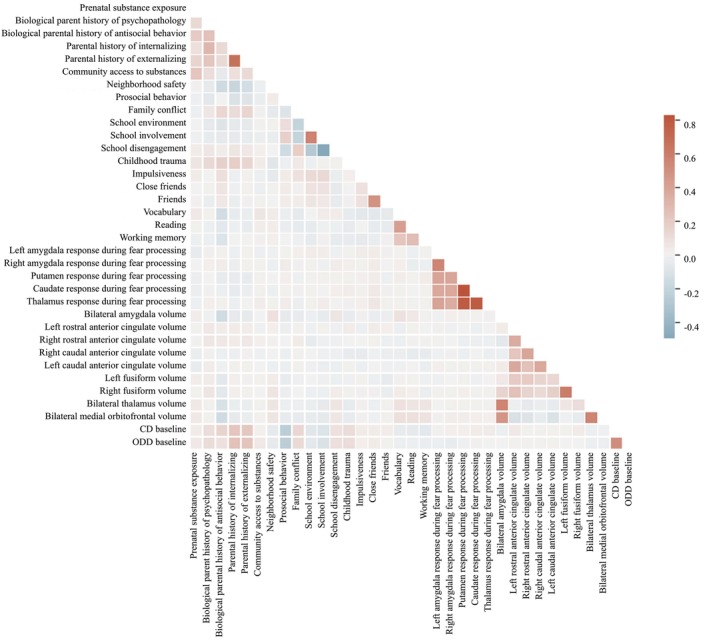
Correlations between variables, used for feature selection

To remove features with duplicate information, we first calculated bivariate correlations between all baseline features; those that were intercorrelated with values >0.7 were inspected (Figure 1). Parental anxiety/depression scores were highly correlated with parent internalizing scores. Parent aggressive behavior, rule‐breaking, antisocial behavior, and externalizing scores were also highly intercorrelated. Therefore, the most predictive features based on Year 1 conduct problem scores were retained using the XGBoost library in Python, which uses a stochastic gradient boosting algorithm to calculate an importance score. Internalizing scores were more predictive than anxiety/depression scores, and externalizing scores were more predictive than aggressive behavior, rule‐breaking, antisocial behavior scores; so these were selected.

Left and right functional ROIs from the N‐back task (i.e., thalamus, caudate, and putamen) were also highly correlated, and so were averaged into a single ROI. Functional activation in bilateral caudate, putamen, and thalamus was also correlated >0.7, so the most predictive feature, the putamen, was selected. Left and right structural ROIs that were correlated bilaterally at >0.7 (amygdala and thalamus) were also each averaged into a single ROI. A figure displaying the feature selection process can be found in the Supporting Information.

#### Evaluating performance

Analyses evaluated how well each classifier was able to predict children's diagnostic status at years 1, 2, and 3 after baseline. To predict conduct problems (clinically significant symptoms of CD or ODD), we calculated the accuracy, precision, and recall of each model (Russell & Norvig, 2021), which reflect the true‐positives, true‐negatives, false‐positives, and false‐negatives generated by each model. Accuracy is calculated as the ratio of correct predictions over the total number of predictions. For example, an accuracy score of .73 would mean that out of 100 participants, the classifier made 73 correct predictions. Model accuracy is useful but was not our key outcome of interest because, in our unbalanced dataset, it would be possible to achieve high accuracy simply by predicting no participants have conduct problems. By contrast, precision is a measure of false diagnosis and is calculated as the ratio of true‐positives over the total number of positive predictions (both true‐ and false‐positives). Finally, recall measures how many times a child with conduct problems was correctly detected. This is measured by the ratio of true‐positives over true‐positives and false‐negatives.

We also used receiver operating characteristics (ROC) curves to calculate the area under the curve (AUC) of each model. The ROC curve plots the sensitivity and specificity of a model for every possible class probability threshold (Fawcett, 2007). Using the classification problem from the current study, sensitivity, or the true‐positive rate, is the probability that the classifier predicts a child has conduct problems when they do in fact have conduct problems. Specificity, or the false‐positive rate, is the probability that the classifier predicts a child has conduct problems when they do not actually have conduct problems. To quantify and compare ROC curves, we calculated the area under the curve. AUC is thought to be a more accurate measure of classifier performance than accuracy, and therefore, we used this as the primary outcome measure to assess the performance of our classifiers (Bradley, 1997).

##### K‐fold cross‐validation

To generate accuracy, precision, recall, and AUC scores, we performed K‐fold cross‐validation with 10‐folds, which is a data resampling technique used to evaluate classifier performance often when the total size of the sample is small (Russell & Norvig, 2021). For classification problems, cross‐validation with 10‐folds has been shown to have minimal bias (Arlot & Celisse, 2010).

#### Feature importance

We also analyzed feature importance in the best performing classifier. These analyses provide a score for each feature entered into the classifier to quantify that feature's predictive value relative to other included features. Features are given a score between 0 and 1 (with all scores in the classifier summing to 1) to signify how much they contribute to classification choice. The higher the score, the more important the feature. The random forest classifier (used because it was later determined to be the best performing classifier) generates a feature importance score using Gini importance (Menze et al., 2009). To create a single value after K‐fold cross‐validation, importance was assessed as the average importance across all folds and was calculated separately for each year.

## Results

### Classifier performance

In general, all models performed well at classifying youths with conduct problems across all 3 years. AUC was highest for the random forest classifier; however, with scores of .98, .97, and .97 for each year, respectively (Table 2). Again, this indicates that the random forest classifier was the most able to detect participants both with and without conduct problems. AUC score for other models was lower, ranging from .88 to .93. We also found accuracy was the highest for the random forest classifier, with accuracy scores of .92, .91, and .92 for each year, respectively, compared with values from the other models that ranged from .69 to .84.

**Table 2:** Accuracy, precision, recall, and area under the curve by year and model type

	Machine learning model					
	Logistic regression	Naïve Bayes	Support vector machine	Random forest	*Simplified random forest*	*Brain random forest*
Accuracy						
Year 1	0.84	0.76	0.80	0.92	*0.91*	*0.82*
Year 2	0.80	0.69	0.75	0.91	*0.90*	*0.85*
Year 3	0.81	0.69	0.78	0.92	*0.90*	*0.84*
Precision						
Year 1	0.83	0.81	0.80	0.92	*0.91*	*0.84*
Year 2	0.79	0.77	0.74	0.92	*0.90*	*0.85*
Year 3	0.81	0.77	0.78	0.92	*0.90*	*0.85*
Recall						
Year 1	0.84	0.79	0.81	0.92	*0.91*	*0.84*
Year 2	0.79	0.74	0.75	0.91	*0.90*	*0.85*
Year 3	0.81	0.74	0.78	0.91	*0.90*	*0.85*
AUC						
Year 1	0.93	0.92	0.91	0.98	*0.97*	*0.94*
Year 2	0.89	0.89	0.86	0.9	*0.96*	*0.93*
Year 3	0.90	0.90	0.88	0.97	*0.97*	*0.93*

*Note:* Italicized MLMs indicate the same Random Forest Classifier trained on different feature subsets (e.g., ten most predictive features [simplified] or brain‐based features [brain]).

The random forest model also had the highest precision, scoring .92 across all years, which suggests a low rate of falsely predicting conduct problems. Finally, the random forest model also had the highest recall, with scores of .92, .91, and .91 for each year, respectively, indicating that this classifier was able to detect children with conduct problems, rarely recording false‐negatives.

### Feature importance

**Figure 2:**
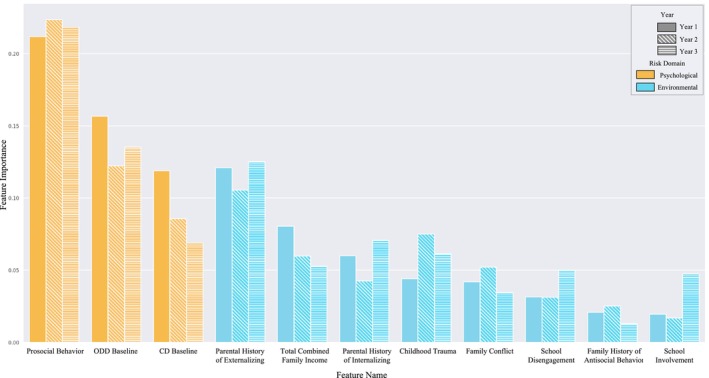
Feature importance scores from the top 10 most important (11 due to variation in feature importance year to year) variables predicting conduct problems after 1, 2, and 3 years in the 39‐item random forest classifier

We next examined the importance of the features within the random forest model. We found that the single most predictive feature was prosocial behavior. This feature was the most relevant variable when predicting the presence of conduct problems across each of the 3 years (Table 3), with feature importance scores of 0.212, 0.223, and 0.219. Notably, the three features with the next highest predictive value across all 3 years (between 0.10 and 0.16 in Year 1) were related to externalizing, including the child's ODD symptoms at baseline, parent externalizing scores, and the child's CD symptoms at baseline (Figure 2). In general, feature importance order remained relatively consistent over the 3‐year period.

**Table 3:** Feature importance in the random forest classifier

	Feature importance		
	Year 1	Year 2	Year 3
Prosocial behavior	0.212	0.223	0.219
ODD baseline	0.157	0.122	0.135
Parental history of externalizing	0.121	0.106	0.125
CD baseline	0.119	0.086	0.069
Total combined family income	0.081	0.060	0.053
Parental history of internalizing	0.060	0.043	0.071
Childhood trauma	0.044	0.075	0.061
Family conflict	0.042	0.052	0.035
School disengagement	0.031	0.031	0.050
Biological parental history of antisocial behavior	0.021	0.025	0.013
School involvement	0.020	0.017	0.047
Neighborhood safety	0.010	0.015	0.006
School environment	0.009	0.010	0.012
Community access to substances	0.009	0.016	0.010
Race/ethnicity	0.006	0.006	0.005
Close friends	0.004	0.005	0.005
Biological parent history of psychopathology	0.004	0.021	0.008
Age	0.003	0.005	0.012
Vocabulary	0.003	0.004	0.003
Left rostral anterior cingulate volume	0.003	0.003	0.003
Sex	0.003	0.007	0.005
Scanner id	0.003	0.009	0.006
Reading	0.003	0.004	0.002
Right fusiform volume	0.003	0.002	0.003
Working memory	0.003	0.003	0.003
Left caudal anterior cingulate volume	0.002	0.004	0.004
Prenatal substance exposure	0.002	0.001	0.002
Left amygdala response during fear processing	0.002	0.005	0.004
Putamen response during fear processing	0.002	0.005	0.002
Friends	0.002	0.005	0.003
Left fusiform volume	0.002	0.005	0.002
Impulsiveness	0.002	0.003	0.002
Right caudal anterior cingulate volume	0.002	0.002	0.004
Right rostral anterior cingulate volume	0.002	0.004	0.003
Intracranial volume	0.002	0.003	0.003
Bilateral thalamus volume	0.002	0.002	0.003
Amygdala volume	0.002	0.003	0.002
Right amygdala response during fear processing	0.002	0.004	0.003
Bilateral medial orbitofrontal volume	0.001	0.003	0.002

In general, in these classifiers, features reflecting brain structure and activation did not have high feature importance (all feature importance scores <0.005).

### Simplified classifier

We next selected the top 10 most important features in the forest classifier separately for each year to assess if a simplified classifier could achieve comparable classification scores. The random forest classifier was retrained on these 10 most important features. This new classifier had scores only slightly lower than those of the more complex classifier trained on 39 rather than 10 features (Table 2). The simplified classifier was similarly accurate (year 1: .91, year 2: .90, year 3: .90) and precise (year 1: .91, year 2: .90, year 3: .90) while also maintaining similar recall (year 1: .91, year 2: .90, year 3: .90) and AUC (year 1: .97, year 2: .96, year 3: .97) scores, indicating that a simplified model is still able to classify participants with and without conduct problems without causing an increase in false diagnosis. Prosocial behavior, ODD risk at baseline, parent externalizing score, CD risk at baseline, and total combined family income remained the most important features when predicting conduct problems at Year 1 (Table 4). Small changes in relative feature importance emerged when predicting conduct problem risk after 2 and 3 years; for example, trauma exposure was more important when predicting outcomes after 2 years. This shortened list of classifiers could prove helpful to clinicians seeking to identify children most in need of interventions.

**Table 4:** Feature importance for the simplified classifier and brain‐based classifier

	Feature importance		
	Year 1	Year 2	Year 3
Simplified model			
ODD baseline	0.244	0.182	0.199
Prosocial behavior	0.234	0.275	0.279
Parental history of externalizing	0.129	0.107	0.115
CD baseline	0.125	0.092	0.078
Parental history of internalizing	0.084	0.048	0.092
Total combined family income	0.069	0.054	0.060
Childhood trauma	0.044	0.147	0.070
Family conflict	0.029	0.032	0.021
Parental history of antisocial behavior	0.022	0.027	
School disengagement	0.019	0.037	0.047
School involvement			0.039
Brain‐based model			
Right caudal anterior cingulate volume	0.038	0.031	0.033
Left rostral anterior cingulate volume	0.038	0.013	0.024
Right rostral anterior cingulate volume	0.018	0.012	0.009
Left caudal anterior cingulate volume	0.017	0.015	0.027
Right fusiform volume	0.017	0.014	0.022
Bilateral medial orbitofrontal volume	0.016	0.029	0.012
Left fusiform volume	0.014	0.073	0.018
Putamen response during fear processing	0.013	0.033	0.008
Intercranial volume	0.010	0.017	0.015
Amygdala volume	0.010	0.024	0.017
Left amygdala response during fear processing	0.008	0.012	0.014
Bilateral thalamus volume	0.007	0.020	0.011
Right amygdala response during fear processing	0.005	0.022	0.028

### Brain‐based classifier

We also trained the random forest classifier on our brain‐based measures while still including all original covariates. This allowed us to assess the classification scores associated with a brain‐based classifier. Compared with classifiers trained on familial and environmental features and psychological features, including the simplified model, this classifier showed lower accuracy (year 1: .82, year 2: .85, year 3: .84), precision (year 1: .84, year 2: .85, year 3: .85), recall (year 1: .84, year 2: .85, year 3: .85), and AUC (year 1: .94, year 2: .93, year 3: .93) scores (Table 2). This indicates that a brain‐based model is able to classify participants with and without conduct problems, but not as well as the optimized classifier that includes a wider range of features. Investigating the features within the brain‐based random forest classifier, we found the single most predictive feature to be right caudal anterior cingulate volume (year 1: .038, year 2: .031, year 3: .033). The second most predictive feature varied based on year and included left rostral anterior cingulate volume in year 1 (.018), left fusiform volume in year 2 (.073), and right amygdala activation during fear processing in year 3 (.028).

## Discussion

Using factors previously linked to conduct problems, we built machine learning models to determine the most robust predictors of conduct problems in children over a 3‐year period. We determined that (1) the most robust classification algorithm, the random forest, could predict which children would exhibit clinically significant conduct problems up to 3 years later with above 90% accuracy, and (2) using this classifier, prosocial behavior, ODD risk at baseline, parent externalizing score, CD risk at baseline, and total family income remained the most consistently important features when predicting risk for conduct problems after 1, 2, and 3 years. These behavioral and environmental features predicted risk for conduct problems beyond biological features (brain structure and function, as measured using MRI). Together, these findings demonstrate the utility of machine learning in feature reduction and identification of at‐risk children, using the outputs of brief, inexpensive self‐report and parent‐report surveys. Using only 10 features, which included child prosocial behavior, baseline child ODD and CD symptoms, and parent history of externalizing, we were able to classify children's risk of persistent, significant conduct problems with over 90% accuracy, precision, and recall.

Notably, the most predictive feature identified by this model was prosocial behavior. The importance of behaviors like sharing or being helpful if someone is upset (Goodman, 1999) is consistent with evidence that callous‐unemotional traits, which include characteristics like reduced empathy and care, are important risk factors for developing conduct problems (Marsh, 2019). In fact, a key feature of callous‐unemotional traits may be that they are associated with not just antisocial behavior, but also reduced prosocial behaviors (Glenn, Efferson, Iyer, & Graham, 2017; Koenigs, Kruepke, & Newman, 2010; Marsh et al., 2014; Viding & McCrory, 2018; Wall, Frick, Fanti, Kimonis, & Lordos, 2016). Together with prior findings, our results suggest that reduced prosocial behaviors may cause worsening social relationships over time, which in turn may lead to more severe conduct problems (Fanti, Mavrommatis, Colins, & Andershed, 2023; Robertson et al., 2023).

Familial and environmental features that predicted conduct problems included parental history of psychiatric disorders, family income, and school involvement. The importance of parental history of psychiatric disorders, both externalizing and internalizing, is consistent with prior research on the development of conduct problems. Longitudinal research has found that parental history of antisocial behavior is a particularly strong predictor of child behavior problems (Murray & Farrington, 2010). Additionally, participants' school engagement or disengagement was specifically predictive of conduct problems, suggesting that a child's attitude toward school, beyond just the school environment itself, plays a significant role in conduct problems. This is consistent with findings that school disengagement increases a child's risk for conduct problems (Vaughn et al., 2011; Wang & Fredricks, 2014), whereas school engagement can act as a protective factor (Barnes, Hoffman, Welte, Farrell, & Dintcheff, 2007; Morrison, Robertson, Laurie, & Kelly, 2002).

Notably, our study's simplified classifier predicting conduct problem risk with reduced features performed nearly as well as the more complex model. A classifier with only 10 features drawn from eight self‐report questionnaires may provide a reasonable tool for early detection of conduct problems. This simplified model excluded brain structure and function variables. This suggests these variables may be less clinically informative than self‐report measures, although identifying biological risk factors for conduct problems remains crucial for understanding underlying mechanisms (Viding & McCrory, 2020). These results align with the broader critique that behavioral measures are often stronger predictors of behavioral outcomes due to inherent challenges in detecting cross‐domain effects (Patrick, 2013).

Study limitations include the relatively low percentage of children at risk for conduct problems due to the focus on healthy child development in the ABCD study (Polanczyk et al., 2015). This necessitated cross‐validation for training and testing. Future research should assess the model's performance across diverse samples, including in clinical settings, to validate its robustness. Also, although the random forest model offers feature importance scores, these scores are relative and may change based on other included features and feature correlations. We sought to mitigate this issue by excluding or consolidating highly correlated features, but stronger correlations among variables likely still influenced feature importance. Another limitation of the current study is the use of emotional n‐back task activation as a proxy for emotion processing. While this task contains an affective manipulation, responses may reflect interactions between memory and emotion rather than emotion alone. Future work would benefit from using latent variable approaches or multiple tasks that more precisely isolate emotion‐related activation. Lastly, another limitation of the current work is that our model does not predict the longitudinal stability or emergence of conduct problems at the individual level across time points. For tables tracking the stability and emergence of conduct problems over time, see the Supporting Information. Future work is needed to assess not only the emergence of new cases but also the persistence or remission of conduct problems over time.

Our study provides evidence that conduct problems in children can be reliably predicted over a 3‐year period using machine learning models incorporating psychological, environmental, and brain‐based features. These results indicate that robust classifiers may not require extensive questionnaires or costly brain‐based measurements. This research also provides guidance for the types of measures that may be important to collect in clinical, pediatric, and school settings to identify children at risk for persistent conduct problems in the hopes that early identification can support early intervention in these settings.

## Ethical considerations

The Adolescent Brain Cognitive Development (ABCD) Study® was reviewed and approved by the Institutional Review Board at the University of California, San Diego (UCSD; IRB #160091, approved September 13, 2016) as the central IRB, with reliance agreements in place across all 21 data collection sites. Each participating site obtained local IRB approval consistent with the master protocol. Written informed consent was obtained from a parent or legal guardian, and assent was obtained from all child participants prior to enrollment.

**Key points**

**What's known**

- Children with conduct problems, such as conduct disorder and oppositional defiant disorder, face elevated risks for negative psychosocial, educational, and behavioral outcomes. Early identification of at‐risk children is crucial for timely intervention and prevention.

**What's new**

- Machine learning classifiers, particularly random forest models, can predict conduct problems with high accuracy (AUC ≥ 0.97) over a 3‐year period using baseline data from children aged 9–10. A simplified model with only 10 features maintained similar performance.

**What's relevant**

- This study demonstrates that parent and child self‐report data combined with machine learning can effectively identify children at risk for persistent conduct problems. These findings highlight the utility of simplified, scalable tools for early screening in clinical and educational settings, promoting targeted interventions.

## Supporting information


**Figure S1.** Feature selection process.
**Table S1.** Retention of participants in the conduct problems group across study waves.
**Table S2.** Emergence of participants in the conduct problems group across study waves.

## Data Availability

The data that support the findings of this study are available from the Adolescent Brain Cognitive Development (ABCD) study. Restrictions apply to the availability of these data, which were used under license for this study. Data are available for request from the National Institute of Mental Health Data Archive at https://nda.nih.gov/abcd/request‐access with the permission of the ABCD Consortium.
