# Supplementary material for: Machine learning prediction of conduct problems in children using the longitudinal ABCD study
Source: J Child Psychol Psychiatry. 2025 Oct 12;67(3):390–9. doi: 10.1111/jcpp.70057 (PMC12883587; doi:10.1111/jcpp.70057)
Supplement: Supplementary file 1 — Figure S1. Feature selection process. Table S1. Retention of participants in the conduct problems group across study waves. Table S2. Emergence of participants in the conduct problems group across study waves. [file JCPP-67-390-s001.docx]

**Supplemental Materials**

***Neural structure and function features***

Functional activation ROIs were selected from the emotional “n-back” task. During this task children viewed faces showing fearful, happy, or neutral expressions drawn from the racially diverse affective expression (RADIATE) face stimulus set (Conley et al., 2018) and images of buildings. Participants were asked each trial to respond if the picture shown is a “Match” or "No Match” to a previous image. In the 2-back condition, participants report "Match” if the current image is the same as the image shown two trials previously. In the 0-back condition participants report “Match” if the current image is the same as the one shown at the start of the task. The task contains only 0 and 2-back conditions. More information about the task and scan protocol are provided in an overview of the ABCD study imaging procedures (Casey et al., 2018).

Following consistent evidence for atypical processing of distress cues like fearful expressions in children with conduct problems (Berluti et al., 2023) we selected functional activation parameters during these trials of the task for inclusion. Mean beta weights during the n-back task were generated and the activation difference from the fearful versus neutral face contrast was calculated. More information about the processing pipeline is reported in an overview of the fMRI analysis pipeline (Hagler et al., 2019).

***Familial and Environmental*** ***Features***

The Adult Self Report was used to assess parent history of psychiatric conditions (Achenbach & Rescorla, 2003). The parent attending the visits reported on their own mental health history. Raw scores from the anxious/depressed, aggressive, rule breaking, internalizing, externalizing, and antisocial personality scales were used. Parents also reported (true or false) if the biological mother or father of their child had a history of manic behavior, hallucinations, delusions, depression, anxiety, suicidal behavior, or have received mental health treatment (0 = no family history; 1 = family history). Parents also reported if the biological mother or father of their child had a history of antisocial behavior including getting into fights or having trouble with the law (0 no family history vs. 1 family history).

Children reported levels of family conflict (e.g., “Family members sometimes get so angry they throw things.”) using the 9 item Conflict subscale from the Family Environment Scale (Moos & Moos, 1994). Items were answered as true (1) or false (0) and assessed the level of conflict within the child’s family and the sum of all items was taken.

Environmental features also included prenatal alcohol and drug exposure measured by parent’s reported known substance use during pregnancy by the child’s biological mother (0 = no use; 1 = use). Substances included tobacco, alcohol, marijuana, Cocaine/Crack, Heroin/Morphine, and Oxycontin.

Children were also asked to report the total number of friends and close friends they have. Close friends were defined as “those you like spending time with, have fun with, and trust”. The maximum allowed value was 100. Number of friends and number of close friends were totaled separately.

Parents reported on access and exposure to drugs and alcohol in their child’s community. The questionnaire assesses the ease at which children are able to access alcohol and drugs on a 4-point Likert like scale from “very hard” (0) to “very easy” (3) and the mean of all reported items was taken. Parent reported neighborhood safety was assessed using the three item Neighborhood Safety Protocol (Echeverria, 2004). Items were on a 5-point Likert like scale ranging from “Strongly Disagree” (1) to “Strongly Agree” (5). A participant’s neighborhood was defined as anything within a 20 minute (about a mile) walk from their home. Items included questions about neighborhood violence and crime (e.g., “Violence is not a problem in my neighborhood”) and the mean of all items was taken.

Children also completed the 13 item School Risk and Protective Factors Survey (Arthur et al., 2007). Three subscales, labelled school environment, school involvement, and school disengagement were calculated by summing the 7, 4, and 2 total items, respectively, to create a score for each subscale. School environment captures a child’s experience of the school and classroom climate (e.g., “My teacher notices when I am doing a good job and lets me know about it”). School involvement captures how much children are positively engaged at school (e.g., “In general, I like school a lot”). School disengagement captures how much children are negatively engaged, or disengaged in school (e.g., “Usually, school bores me”).

Parents and child reports were used to calculate Adverse Childhood Experience (ACEs) scores, including only variables collected at baseline (Stinson et al., 2021). ACE scores were summed from parent and child reported adverse events including abuse (emotional, physical, and sexual), neglect (emotional, and physical), negative family events (parental separation/divorce and family member involvement with legal system), and other significant adverse experiences. Scores did not include Mental Illness in Household as that feature was separately included in the model.

***Psychological Features***

Psychological features included variables related to cognitive and psychosocial traits. These included three subscale scores assessing children’s cognitive ability, drawn from the NIH toolbox, which was administered in a computerized format (Gershon et al., 2013). The toolbox included the Picture Vocabulary test assessing vocabulary, Oral Reading Recognition test assessing reading ability, and the List Sorting Working Memory test assessing working memory. Fully corrected T-scores were used.

Variables related to impulsiveness were also included, and were drawn from the child-completed Urgency, Premeditation, Perseverance, Sensation Seeking, Positive Urgency, Impulsive Behavior Scale (UPPS-P) Short Form (ABCD-version). Items (e.g., “I enjoy taking risks”) were scored on a 4-point scale ranging from 1, “Not at all like me”, to 4, “Very much like me”. The mean of all 20 items was taken.

Positive social functioning variables were also included. Items were drawn from the prosocial behavior subscale from the Strengths and Difficulties Questionnaire (i.e., “My child is considerate of other people's feelings.”, “Helpful if someone is hurt, upset, or feeling ill.”, “Often offers to help others (parents, teachers, other children).”) (Goodman, 1999). Parents reported their child’s prosocial behavior using a 3-point scale, labelled “Not True” (0), “Somewhat True” (1), or “Certainly True” (2) Scores were calculated as the mean of these items.

**Demographic Features**

Biological sex at birth (0 = male; 1 = female), race/ethnicity (1 = White; 2 = Black; 3 = Hispanic; 4 = Asian; 5 = Other), total combined family income in the past 12 months, and age (in months) were included as demographic features.

**Covariates**

Scanner ID, data collection site, total intercranial volume, and baseline Conduct Disorder (0 = no diagnosis; 1 = diagnosis risk) and Oppositional Defiant Disorder diagnosis (0 = no diagnosis; 1 = diagnosis risk) were included as covariates in all classifiers. Details of all features and the feature selection process are depicted in Supplementary Figure 1.

***Missing Data Imputation***

During the imputation process, auxiliary variables were included to ensure that data was plausibly missing at random (i.e., parent age, parent employment status, parent marital status, parent’s partner’s employment status, number of people living at their address, child’s time spent at another house, parent’s biological relationship to child) (Azur et al., 2011; White et al., 2011; Wulff & Jeppesen, 2017). Data were normalized using min/max scaling in order to improve training speed (Wulff & Jeppesen, 2017). Due to the imbalanced nature of the dataset (only 5-7% of participants had clinically significant conduct problems) we resampled the data to create more equal classes because it is important to train classification algorithms on datasets with roughly equivalent numbers of positive and negative cases (i.e., at risk, not at risk). We used the sampling algorithm SMOTE-ENN which combines synthesizing minority oversampling technology (SMOTE) and the Wilson’s Edited Nearest Neighbor Rule (ENN) (Batista et al., 2004). This method reduces the possibility of overfitting while over-sampling our minority class.

**Stability of Conduct Problems**

In order to better understand the stability and emergence of conduct problems over time we evaluated changes in conduct problem status across study waves. We found variation in conduct problems group membership over time. The number of participants with conduct problems at baseline who remained in the conduct problem group in all three subsequent waves was relatively small, making up 22.45% of the original sample (Supplemental Table 1). Additionally, in a notable proportion of participants, conduct emerged after the first wave of data collection (Supplemental Table 2).

**Supplemental Figure 1. Feature Selection Process**

| **Category** | **Features** | **Feature Selection Outcome** |
| --- | --- | --- |
| Familial and Environmental | Prenatal Substance Exposure | Prenatal Substance Exposure |
|  | Parental History of Psychopathology | Parental History of Psychopathology |
|  | Parental History of Antisocial Behavior | Parental History of Antisocial Behavior |
|  | Parental History of Internalizing Symptoms | Parental History of Internalizing Symptoms |
|  | Parental Anxiety/Depression | Dropped For: Parental History of Internalizing Symptoms |
|  | Parental History of Externalizing Symptoms | Parental History of Externalizing Symptoms |
|  | Parental Aggressive Behavior | Dropped For: Parental History of Externalizing Symptoms |
|  | Parental Rule-Breaking Behavior | Dropped For: Parental History of Externalizing Symptoms |
|  | Parental Antisocial Behavior | Dropped For: Parental History of Externalizing Symptoms |
|  | Community Access to Substances | Community Access to Substances |
|  | Neighborhood Safety | Neighborhood Safety |
|  | Family Conflict | Family Conflict |
|  | School Environment Quality | School Environment Quality |
|  | School Involvement | School Involvement |
|  | School Disengagement | School Disengagement |
|  | Childhood Trauma | Childhood Trauma |
|  | Close Friendships | Close Friendships |
|  | Friendships | Friendships |
| Psychological | Prosocial Behavior | Prosocial Behavior |
|  | Impulsivity | Impulsivity |
|  | Vocabulary Skills | Vocabulary Skills |
|  | Reading Ability | Reading Ability |
|  | Working Memory | Working Memory |
| Neural structure and function | Left Amygdala Activation During Fear Processing | Left Amygdala Activation During Fear Processing |
|  | Right Amygdala Activation During Fear Processing | Right Amygdala Activation During Fear Processing |
|  | Left Putamen Activation During Fear Processing | Dropped For: Thalamus Activation During Fear Processing |
|  | Right Putamen Activation During Fear Processing | Dropped For: Thalamus Activation During Fear Processing |
|  | Left Caudate Activation During Fear Processing | Dropped For: Thalamus Activation During Fear Processing |
|  | Right Caudate Activation During Fear Processing | Dropped For: Thalamus Activation During Fear Processing |
|  | Left Thalamus Activation During Fear Processing | Mereged: Thalamus Activation During Fear Processing |
|  | Right Thalamus Activation During Fear Processing |  |
|  | Left Amygdala Volume | Mereged: Amygdala Volume |
|  | Right Amygdala Volume |  |
|  | Left Rostral Anterior Cingulate Volume | Left Rostral Anterior Cingulate Volume |
|  | Right Rostral Anterior Cingulate Volume | Right Rostral Anterior Cingulate Volume |
|  | Left Caudal Anterior Cingulate Volume | Left Caudal Anterior Cingulate Volume |
|  | Right Caudal Anterior Cingulate Volume | Right Caudal Anterior Cingulate Volume |
|  | Left Fusiform Gyrus Volume | Left Fusiform Gyrus Volume |
|  | Right Fusiform Gyrus Volume | Right Fusiform Gyrus Volume |
|  | Left Thalamus Volume | Merged: Thalamus Volume |
|  | Right Thalamus Volume |  |
|  | Left Medial Orbitofrontal Cortex Volume | Merged: Medial Orbitofrontal Cortex Volume |
|  | Right Medial Orbitofrontal Cortex Volume |  |
| Demographic | Age | Age |
|  | Gender | Gender |
|  | Race/Ethnicity | Race/Ethnicity |
|  | Total Combined Family Income | Total Combined Family Income |
| Covariates | Conduct Disorder (CD) Baseline | Conduct Disorder (CD) Baseline |
|  | Oppositional Defiant Disorder (ODD) Baseline | Oppositional Defiant Disorder (ODD) Baseline |
|  | Scanner ID | Scanner ID |
|  | Intracranial Volume | Intracranial Volume |
|  | Data Collection Site | Data Collection Site |

**Supplemental Table 1. Retention of Participants in the Conduct Problems Group Across Study Waves**

| **Study Wave** | **N** |
| --- | --- |
| Baseline | 245 |
| Baseline and Year 1 | 134 |
| Baseline, Year 1, and Year 2 | 77 |
| Baseline, Year 1, Year 2, and Year 3 | 55 |

Note: We include the baseline count for reference. There is no data before baseline and therefore we cannot know what proportion of participants in the baseline group had conduct problems prior to baseline.

**Supplemental Table 2. Emergence of Participants in the Conduct Problems Group Across Study Waves**

| **Study Wave** | **N** |
| --- | --- |
| Baseline | 245 |
| Year 1 | 100 |
| Year 2 | 79 |
| Year 3 | 55 |

Note: We include the baseline count for reference. There is no data before baseline and therefore we cannot know what proportion of participants in the baseline group had an emergence of conduct problems.

References

Azur, M. J., Stuart, E. A., Frangakis, C., & Leaf, P. J. (2011). Multiple imputation by chained equations: what is it and how does it work? *International Journal of Methods in Psychiatric Research*, *20*(1), 40–49. https://doi.org/10.1002/mpr.329

Batista, G. E. A. P. A., Prati, R. C., & Monard, M. C. (2004). A study of the behavior of several methods for balancing machine learning training data. *ACM SIGKDD Explorations Newsletter*, *6*(1), 20–29. https://doi.org/10.1145/1007730.1007735

Berluti, K., Ploe, M. L., & Marsh, A. A. (2023). Emotion processing in youths with conduct problems: an fMRI meta-analysis. *Translational Psychiatry*, *13*(1), 105. https://doi.org/10.1038/s41398-023-02363-z

Casey, B. J., Cannonier, T., Conley, M. I., Cohen, A. O., Barch, D. M., Heitzeg, M. M., Soules, M. E., Teslovich, T., Dellarco, D. V., Garavan, H., Orr, C. A., Wager, T. D., Banich, M. T., Speer, N. K., Sutherland, M. T., Riedel, M. C., Dick, A. S., Bjork, J. M., Thomas, K. M., … Dale, A. M. (2018). The Adolescent Brain Cognitive Development (ABCD) study: Imaging acquisition across 21 sites. *Developmental Cognitive Neuroscience*, *32*, 43–54. https://doi.org/10.1016/j.dcn.2018.03.001

Conley, M. I., Dellarco, D. V., Rubien-Thomas, E., Cohen, A. O., Cervera, A., Tottenham, N., & Casey, B. (2018). The racially diverse affective expression (RADIATE) face stimulus set. *Psychiatry Research*, *270*, 1059–1067. https://doi.org/10.1016/j.psychres.2018.04.066

Gershon, R. C., Slotkin, J., Manly, J. J., Blitz, D. L., Beaumont, J. L., Schnipke, D., Wallner-Allen, K., Golinkoff, R. M., Gleason, J. B., Hirsh-Pasek, K., Adams, M. J., & Weintraub, S. (2013). IV. NIH TOOLBOX COGNITION BATTERY (CB): MEASURING LANGUAGE (VOCABULARY COMPREHENSION AND READING DECODING). *Monographs of the Society for Research in Child Development*, *78*(4), 49–69. https://doi.org/10.1111/mono.12034

Goodman, R. (1999). The Extended Version of the Strengths and Difficulties Questionnaire as a Guide to Child Psychiatric Caseness and Consequent Burden. *Journal of Child Psychology and Psychiatry*, *40*(5), 791–799. https://doi.org/10.1111/1469-7610.00494

Hagler, D. J., Hatton, SeanN., Cornejo, M. D., Makowski, C., Fair, D. A., Dick, A. S., Sutherland, M. T., Casey, B. J., Barch, D. M., Harms, M. P., Watts, R., Bjork, J. M., Garavan, H. P., Hilmer, L., Pung, C. J., Sicat, C. S., Kuperman, J., Bartsch, H., Xue, F., … Dale, A. M. (2019). Image processing and analysis methods for the Adolescent Brain Cognitive Development Study. *NeuroImage*, *202*, 116091. https://doi.org/10.1016/j.neuroimage.2019.116091

Stinson, E. A., Sullivan, R. M., Peteet, B. J., Tapert, S. F., Baker, F. C., Breslin, F. J., Dick, A. S., Gonzalez, M. R., Guillaume, M., Marshall, A. T., McCabe, C. J., Pelham, W. E., Van Rinsveld, A., Sheth, C. S., Sowell, E. R., Wade, N. E., Wallace, A. L., & Lisdahl, K. M. (2021). Longitudinal Impact of Childhood Adversity on Early Adolescent Mental Health During the COVID-19 Pandemic in the ABCD Study Cohort: Does Race or Ethnicity Moderate Findings? *Biological Psychiatry Global Open Science*, *1*(4), 324–335. https://doi.org/10.1016/j.bpsgos.2021.08.007

White, I. R., Royston, P., & Wood, A. M. (2011). Multiple imputation using chained equations: Issues and guidance for practice. *Statistics in Medicine*, *30*(4), 377–399. https://doi.org/10.1002/sim.4067

Wulff, J. N., & Jeppesen, L. E. (2017). Multiple imputation by chained equations in praxis: Guidelines and review. *Electronic Journal of Business Research Methods*, *15*(1), 41–56.
